# Women with endometriosis improved their peripheral antioxidant markers after the application of a high antioxidant diet

**DOI:** 10.1186/1477-7827-7-54

**Published:** 2009-05-28

**Authors:** Jennifer Mier-Cabrera, Tania Aburto-Soto, Soraya Burrola-Méndez, Luis Jiménez-Zamudio, Mari C Tolentino, Esther Casanueva, César Hernández-Guerrero

**Affiliations:** 1 Departamento de Biología Celular, Instituto Nacional de Perinatología "Isidro Espinosa de los Reyes", Mexico City, Mexico; 2 Departamento de Salud, Universidad Iberoamericana Campus Santa Fe, Mexico City, Mexico; 3 Laboratorio de Inmunología Clínica I, Departamento de Inmunología, Escuela Nacional de Ciencias Biológicas, Instituto Politécnico Nacional, Mexico City, Mexico; 4 Subdirección de Investigación en Salud Pública, Instituto Nacional de Perinatología "Isidro Espinosa de los Reyes", Mexico City, Mexico

## Abstract

**Background:**

Oxidative stress has been identified in the peritoneal fluid and peripheral blood of women with endometriosis. However, there is little information on the antioxidant intake for this group of women. The objectives of this work were 1) to compare the antioxidant intake among women with and without endometriosis and 2) to design and apply a high antioxidant diet to evaluate its capacity to reduce oxidative stress markers and improve antioxidant markers in the peripheral blood of women with endometriosis.

**Methods:**

Women with (WEN, n = 83) and without endometriosis (WWE, n = 80) were interviewed using a Food Frequency Questionnaire to compare their antioxidant intake (of vitamins and minerals). Then, the WEN participated in the application of a control (n = 35) and high antioxidant diet (n = 37) for four months. The high antioxidant diet (HAD) guaranteed the intake of 150% of the suggested daily intake of vitamin A (1050 μg retinol equivalents), 660% of the recommended daily intake (RDI) of vitamin C (500 mg) and 133% of the RDI of vitamin E (20 mg). Oxidative stress and antioxidant markers (vitamins and antioxidant enzymatic activity) were determined in plasma every month.

**Results:**

Comparison of antioxidant intake between WWE and WEN showed a lower intake of vitamins A, C, E, zinc, and copper by WEN (p < 0.05, Mann Whitney Rank test). The selenium intake was not statistically different between groups. During the study, the comparison of the 24-hour recalls between groups showed a higher intake of the three vitamins in the HAD group. An increase in the vitamin concentrations (serum retinol, alpha-tocopherol, leukocyte and plasma ascorbate) and antioxidant enzyme activity (superoxide dismutase and glutathione peroxidase) as well as a decrease in oxidative stress markers (malondialdehyde and lipid hydroperoxides) were observed in the HAD group after two months of intervention. These phenomena were not observed in the control group.

**Conclusion:**

WEN had a lower intake of antioxidants in comparison to WWE. Peripheral oxidative stress markers diminished, and antioxidant markers were enhanced, in WEN after the application of the HAD.

## Background

Endometriosis is a gynecological disorder characterized by the presence and growth of endometrial tissue outside the uterine cavity [1]. The prevalence of endometriosis among asymptomatic women ranges from 2–22%, while in women with infertility the incidence of endometriosis is 35% to 50% [2]. This pathology represents one of the most frequent gynecological disorders during women's reproductive age. The most common theory to explain the presence of endometrial tissue outside of the endometrial layer is retrograde menstruation, which consists of the reflux of menstrual fluid through the Fallopian tubes to the abdominal cavity (Sampson's Theory) [3].

Data reported in the literature have suggested that oxidative stress might play a role in the development and progression of endometriosis [4]. Evidence shows a higher concentration of lipid peroxidation markers in the peripheral blood and peritoneal fluid of women with endometriosis. These higher concentrations might increase growth and adhesion of endometrial cells in the peritoneal cavity [5,6]. The higher lipid peroxidation markers observed in women with endometriosis have been related to a pro-inflammatory milieu, a consequence of the activation of peritoneal macrophages due to the presence of apoptotic and undigested endometrial ectopic cells and red blood cells [7]. Upon activation, peritoneal macrophages release reactive oxygen and nitrogen species (RONS), also known as free radicals. Studies of the peritoneal fluid have confirmed an increased number, concentration and activation of macrophages in women with this disorder in comparison with women without endometriosis [8-10].

Abnormally high concentrations of free radicals could disrupt the balance between oxidants and antioxidants, promoting oxidative stress conditions. Oxidative stress develops if the production of RONS is increased and/or the level of antioxidants is decreased [11]. Our defenses against RONS damage are the antioxidants synthesized in the body and taken up in the diet. These enzymatic and non-enzymatic antioxidant defense systems allow scavenging of RONS. The former include proteins (e.g., catalase, glutathione peroxidase and superoxide dismutase) that need a small concentration of minerals to show their optimal enzymatic activity. The latter (ascorbate, α-tocopherol, β-carotene and minerals) need to be eaten regularly because they generally do not accumulate in the body [12].

At present, the investigation related to the oxidative stress shown by this group of women is focused on the characteristics that promote oxidative conditions (e.g., peritoneal proinflammatory cytokine synthesis, peritoneal mononuclear cell activation and phagocyte respiratory burst) [8,11,13,14]. However, there is little information on the consumption of antioxidants (vitamins and minerals that may counter oxidative stress) in women with endometriosis [15] and on the condition's possible relationship with oxidative stress [16].

The objective of the present work was to compare the antioxidant intake among women with and without endometriosis and the design and application of a high-antioxidant diet (vitamins A, C and E) to evaluate its capacity to abrogate oxidative stress markers and improve antioxidant markers in the plasma of women with endometriosis.

## Methods

### Setting

The study was carried out at the Instituto Nacional de Perinatología "Isidro Espinosa de los Reyes" (INPerIER) in Mexico City.

### Antioxidant intake in women with and without endometriosis

Mexican mestizo women from the "altiplano central" without (n = 80) and with endometriosis (n = 86) were recruited by means of their medical records; each record was checked one day before the surgery to make sure the women fulfilled the inclusion criteria for each group. The day after the surgery, women who elected to participate gave their informed and written consent and were asked for demographic, socioeconomic and educational status. The INPerIER's academic-ethic bureau approved this research protocol (212250-06081). The study procedures are in accordance with the Helsinki Declaration of Human Rights.

The group of women without endometriosis (WWE) included fertile women with satisfied parity who came to INPerIER for tubal ligation as a permanent contraceptive method; absence of endometriosis was confirmed during surgery. The women with endometriosis (WEN) group were infertile women patients at INPerIER's Infertility Clinic who underwent diagnostic laparoscopy for infertility and were confirmed to have I- or II- stage endometriosis according to the revised criteria of the American Society for Reproductive Medicine (r-ASRM) [17]. The exclusion criteria for both groups were: the presence and diagnosis of inflammatory pelvic disease, a previous history of autoimmune or endocrine/metabolic diseases, tobacco and alcohol consumption, and/or use of multivitamin supplements.

A trained nutritionist, who did not know the fertility status of the subjects, assessed the dietary intake of women without and with endometriosis using a Food Frequency Questionnaire (FFQ) for Mexican women [18]. Antioxidant intake was obtained using the SNUT software ("Sistema de Evaluación de Hábitos Nutricionales y Consumo de Alimentos", Instituto Nacional de Salud Pública, México), and values were reported as a percentage of the Recommended Daily Intake (% RDI).

### Antioxidant supplementation in women with endometriosis

All WEN (n = 83) participated in the application of a high antioxidant diet (HAD). WEN were randomly assigned to group 1 (normal diet) or group 2 (HAD) in their first. There were five visits for each patient. Each visit included anthropometric measurements: height (only at the beginning of the study) using a digital stadiometer (SECA model 242, Hamburg, Germany), weight and calculation of body mass index (Bioimpedometer Tanita model TBF-300A, Tokyo, Japan). Patients were eliminated if they either missed two follow-up visits or became pregnant during the diet study. The normal diet or the HAD was tailored according to each patient's energy requirements. Energy requirements (required daily caloric intake) were estimated on the basis of body weight, age, and physical activity using the Mifflin-St. Joer equation, which gave the most reliable results [19]. A measuring cup was given in order to standardize the food portions subjects would consume.

For 4 months, patients returned for a follow-up visit every 30 days and filled out a multiple-step 24-hour recall [20] to evaluate antioxidant vitamin intake. Food models were used (Life/form^® ^Replica, NASCO, USA) to estimate portion sizes. Retinol, ascorbic acid and α-tocopherol intakes were calculated using the Food Processor Nutrition Analysis software version 8.3.0 (Esha, Oregon, USA).

### Design of the high antioxidant diet

A high antioxidant diet (HAD) was designed based on the "Sistema Mexicano de Equivalentes" [21]. Three food groups were created in order to guarantee 150% of the intake of the Suggested Daily Intake (SDI) of vitamin A (1050 μg retinol equivalent), 660% of the Recommended Daily Intake (RDI) of vitamin C (500 mg) and 133% of the RDI of vitamin E (20 mg) for Mexican women [22]. The groups were named as follows: "Vegetables A", "Fruit A" and "Seeds", respectively.

The "Vegetables A" and "Fruit A" groups included foods with a high content of vitamins A and C, respectively [23]. Estimations were done to reach a supply of 300 μg of retinol (vegetables) and 100 mg of vitamin C (fruit) (Table 1). Vitamin E-rich foods constituted the "Seeds" group. Sunflower seeds and peanuts supplied this vitamin at a low cost. Four tablespoons of sunflower seeds and three tablespoons of peanuts supply 16.75 mg and 3.25 mg of α-tocopherol, respectively.

**Table 1:** "Vegetable A" and "Fruit A" groups; standardized food portions that supply 300 μg of retinol^1 ^(150% SDI) and 100 mg of vitamin C^1 ^(660% RDI).

Vegetables "A"	Net Weight (g)	Vitamin A (μgER)	Vitamin C (mg)	Energy (Kcal)	Carbs (g)	Prot (g)	Fat (g)	Example^2^
Boiled swiss chard	14	300	81	20.0	3.6	2.2	0.2	1/3 cup
Boiled broccoli	18	300	96	57.5	10.6	6.5	0.5	3/4 cup
Boiled spinach	5	300	48	15.0	1.6	2.7	0.4	1/2 cup
Mexican pepperleaf	25	300	162	24.1	3.7	1.9	0.8	1/4 cup
Tomato	13	300	57	11.2	2.5	0.4	0.1	1/2 piece
Edible cactus leaves	17	300	81	31.2	6.5	2.0	0.3	3/4 cup
Lambsquarters	15	300	96	20.0	3.2	2.4	0.3	1/3 cup
Romeritos	15	300	84	27.0	4.7	3.5	0.2	1/2 cup
Purslane	15	300	78	28.0	5.3	2.5	0.3	3/4 cup
Carrot	32	300	132	19.8	4.7	0.2	0.1	1/2 cup
**Mean**	-	300	92	25.4	4.6	2.4	0.3	-
**SD**	-	0	34	12.8	2.5	1.7	0.2	-
								
**FRUIT "A"**								
Strawberries	114	0.8	100	52.6	12.3	1.1	0.7	15 pieces
Guava	366	1.2	100	27.9	6.5	0.4	0.3	1/2 piece
Kiwi	196	0.8	100	62.2	15.2	1.0	0.4	1 piece
Lemon	154	0.6	100	26.0	13.9	1.6	0.4	5 pieces
Cantaloupe	84	0.6	100	66.7	15.7	1.7	0.7	1 cup
Orange	106	0.2	100	88.7	22.3	1.7	0.2	2 pieces
Orange juice	100	0.4	100	90.0	20.8	1.4	0.4	1/2 cup
Papaya	124	0.2	100	62.9	15.8	1.0	0.2	1 cup
Grapefruit	106	0.8	100	86.8	20.9	1.5	0.8	1 piece
Black sapotas	166	0.2	100	67.5	17.5	1.0	0.1	1/2 cup
**Mean**	-	0.58	100	63.1	16.1	1.2	0.4	-
**SD**	-	17.222	0	22.9	4.7	0.4	0.2	-

^1 ^According to the "Tablas de valor nutritivo de los alimentos"[21].

^2 ^The proposed SDI for vitamin A and RDI for vitamin C were obtained by the intake of three portions of "Vegetables A" group and five portions of "Fruit A" group.

Diets with different caloric densities were 55-15-30 (55% calories from carbohydrates, 15% from protein and 30% from dietary fat). The proposed SDI for vitamin A and the RDIs for vitamins C and E were obtained by the intake of three portions of "Vegetables A" group, five portions of "Fruit A" group, and the established portion for "Seeds", which altogether supplied 2403 μg of copper (320% RDI), 41.8 μg of selenium (87% RDI) and 5.5 mg of zinc (50% RDI).

### Blood samples

Every follow-up visit, blood samples were drawn by vein puncture in heparin, EDTA and no-additive tubes (Becton Dickinson, Vacutainer Systems, New Jersey, USA). Samples were placed on ice and taken immediately to the laboratory for processing according to the requirements for every method employed. Plasma and serum samples were stored at -70°C with butylated hydroxytoluene (Sigma, Missouri, USA), as a free radical scavenger, at a final concentration of 20 μmol/L.

### Oxidative stress determination

Total plasma lipid hydroperoxides (LHP) was determined using the ferrous oxidation in Xylenol Orange (FOX) assay [24]. The method is based on the principle of the rapid peroxide-mediated oxidation of Fe^2+ ^to Fe^3+ ^under acidic conditions. The latter, in the presence of xylenol orange, forms a Fe^3+^-xylenol orange complex that can be measured spectrophotometrically at 560 nm. A standard curve of H_2_O_2 _from 0 to 100 μM was used.

Malondialdehyde (MDA) was estimated by measurement of thiobarbituric acid reactive substances (TBARS) [25]. The pink chromogen produced was measured spectrophotometrically at 530 nm. A standard curve of MDA in concentrations from 0 to 0.7 μM was used.

All reagents used were from Sigma (Missouri, USA). The coefficients of variation for intra- and inter-assays in lipid hydroperoxides determination were 3.5% and 7.5%, respectively, and 4.5% and 8%, respectively, in malondialdehyde.

### Measurement of vitamins in serum, plasma and leukocytes

Plasma and leukocyte ascorbate was analyzed by paired-ion, reversed-phase HPLC coupled with electrochemical detection (Perkin Elmer Instruments, California, USA) [26]. The chromatographic conditions were optimized to give a maximum separation with minimal elution time using a C18 reversed-phase column, with a mobile phase of sodium acetate buffer (pH 4) containing n-octylamine (1 mM) as the paired-ion reagent at a flow rate of 1 mL/min. Ascorbic acid was detected with the electrochemical detector pre-set at 0.7 V and current at 100 mA/V.

Serum retinol and α-tocopherol were measured by reversed-phase HPLC (Perkin Elmer Instruments, California, USA) [27]. Proteins in the serum were denatured with ethanol. The carotenoids and tocopherols were extracted with hexane, and the analysis was performed using a C18 reversed-phase column with an isocratic mobile phase. Analysis of the carotenoids utilized a diode array detector, and analysis of tocopherols used fluorescence. Retinil acetate and α-tocopherol acetate were used as internal standards. The coefficients of variation inter- and intra-assays were less than 5% and 8%, respectively, in all determinations.

### Enzyme activities

Superoxide dismutase (SOD) activity was measured in plasma using a tetrazolium salt for detection of superoxide radicals generated by xanthine oxidase and hypoxanthine. The chromophore produced has a maximal absorbance at 525 nm. One unit of SOD is defined as the amount of enzyme needed to exhibit 50% dismutation of the superoxide radical.

Glutathione peroxidase (GPx) activity was measured using a kinetic colorimetric assay that measures activity indirectly by a coupled reaction with glutathione reductase. Glutathione reductase and NADPH reduce oxidized glutathione. NADPH oxidation is accompanied by a decrease in absorbance at 340 nm, and the decrease is directly proportional to the GPx activity in the sample.

Both enzymatic activities were measured using commercially available assay kits (Cayman Chemical, Michigan, USA) following the methodology described by the manufacturer.

### Statistical analysis

Data are presented as mean ± SD. Food intake (24-hr recalls), and the effects of control and HAD on biochemical determinations were analyzed by Friedman Repeated Measures Analysis of Variance on Ranks following Dunn's post hoc test. Age, body mass index (BMI) and body fat percentages were analyzed by Student's paired t-test. Antioxidant vitamin and mineral intake, as well as the comparison of data between the normal diet and the HAD, was analyzed by Mann-Whitney U-Rank Sum test (non-parametric data). A p < 0.05 was considered statistically different. The SigmaStat software version 3.11 (Systat Software Inc., California, USA) was used to perform all statistical analyses.

## Results

### Women without vs. with endometriosis: general characteristics

All women with and without endometriosis who participated in this study lived in the metropolitan area of Mexico City and had low educational and socio-economical status. No differences were found between groups (data not shown).

The mean age of WWE was 33.8 ± 3.15, which was not statistically different from 32.70 ± 2.46 years from the WEN group (p > 0.05, Student's t test). The obstetric characteristics of WWE (median (min-max)) were the following: gravidity = 3 (1–7), vaginal delivery = 1 (0–4), Cesarean delivery = 1 (1–2) and abortion = 0 (0–2). On the other hand, WEN were nulligravida (p < 0.05, Mann-Whitney comparison WWE vs. WEN in all cases).

### Women with endometriosis: diet study

General characteristics of women with endometriosis in the normal diet (n = 35) and HAD groups (n = 37) are described in table 2. Five women dropped out from the normal diet group due to lack of interest (n = 2) and missing two follow-up visits (n = 3). Meanwhile, three women from the HAD group left the study for personal reasons (n = 1) and missing two follow-up visits (n = 2).

**Table 2:** General characteristics of women with endometriosis in the control and HAD groups.

	Control n = 35	HAD n = 37
Completed the study (%)	35/40 (88)	37/40 (93)
Age (years)	30.8 ± 2.4	31.3 ± 3.4
BMI (Kg/m^2^)		
Basal	24.61 ± 3.02	24.88 ± 2.99
Final	24.14 ± 3.02*	24.49 ± 3.10*
Overweight-Obesity prevalence^1 ^(%)		
Basal	19/35 (54)-1/35 (3)	20/37 (54)-1/37 (3)
Final	15/35 (43)-1/35 (3)	15/37 (40)-1/37 (3)
Fat (%)		
Basal	29.40 ± 5.13	29.78 ± 5.64
Final	28.09 ± 5.06*	28.78 ± 5.91*
Obesity prevalence^2 ^(%)		
Basal	14/35 (40)	15/37 (41)
Final	10/35 (28)	11/37 (30)

Data are means ± SD unless noted. Age, BMI and % Fat were analyzed by Student's paired t-test.

^1 ^Overweight and obesity values within the range 25.0–29.9 and 30.0–34.9, according to the WHO.

^2 ^Considered as obesity women with % fat values above 30%.

*P < 0.05 based on a Student's paired t-test (basal vs. final values).

### Women without vs. with endometriosis: antioxidant intake

The FFQ reported that in the WWE group, the intakes of vitamin C (75 mg = 100% RDI) and copper (750 μg = 100% RDI) were 446% RDI and 418%RDI, respectively. Meanwhile, intakes of vitamin A (570 μg retinol equivalents = 100% SDI) and zinc (11 mg = 100%) were 163%SDI and 178%RDI, respectively. Vitamin E intake (13 mg = 100% RDI) was 112%RDI. Only selenium (48 mg = 100% RDI) intake was below the recommended value for Mexican women and did not meet its 100% RDI.

The WEN group intake of vitamin C and copper was 308%RDI and 299%RDI, respectively. The vitamin A and zinc intake was 110%SDI and 122% RDI, respectively. Vitamin E and selenium intake was 66% and 58% RDI, respectively. This is below their recommended values, so their RDIs were not met.

When antioxidant intake in WEN (n = 83) was compared to the antioxidant intake in WWE (n = 80), vitamin A, vitamin E, vitamin C, zinc, and copper intake was lower in the endometriotic group and showed a statistical difference (p < 0.05, Mann Whitney Rank-U test). The selenium intake was not statistically different between groups (p > 0.05) (Table 3).

**Table 3:** Antioxidant vitamin and mineral intake in women without and with endometriosis.

Nutrient	Women without endometriosis (n = 83)	Women with endometriosis (n = 80)
Vitamin A	163 ± 45 (165; 101–270)	110 ± 23* (104; 87–197)
Vitamin C	446 ± 142 (431; 150–909)	308 ± 162* (276; 109–871)
Vitamin E	112 ± 26 (114; 61–100)	66 ± 27* (60; 23–133)
Copper	418 ± 97 (417; 229–679)	299 ± 57* (316; 211–380)
Zinc	178 ± 62 (166; 79–307)	122 ± 40* (123; 50–231)
Selenium	63 ± 20 (62; 34–114)	58 ± 24 (54; 26–130)

Means ± SD (median; min-max). Data are expressed as percentage of RDI values.

* p < 0.05, Mann Whitney Rank-U test. WWE vs. WEN.

### Women with endometriosis: antioxidant vitamin intake during the diet study by 24-hr recalls

**Figure 1:**
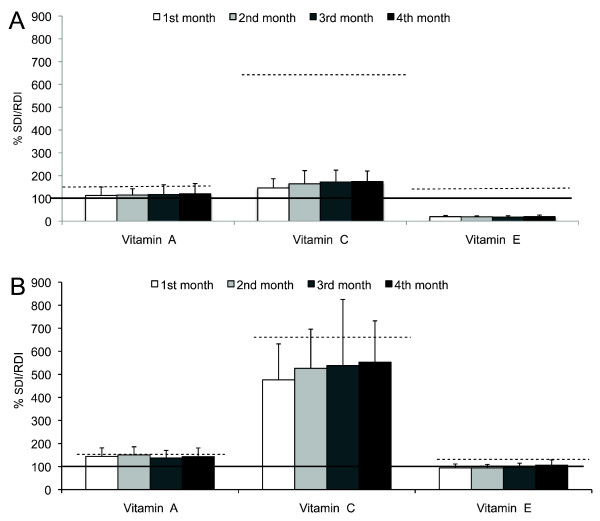
**24-hr recalls in the control and HAD groups**. Vitamin A, C and E intake during the four months of intervention in the (A) control and (B) HAD groups. Continuous line (—) represents the 100% of the SDI or RDI of the vitamins. Dashed lines (---) represent the intakes proposed for the study (vitamin A: 150%; vitamin C: 660%; vitamin E: 133%). Data are expressed as percentage of SDI or RDI and bars represent mean ± SD.

Results showed no statistical differences in the intake of vitamin A, vitamin C and vitamin E in the control and in the HAD groups during the study's duration (Figure 1). Nevertheless, the comparison of monthly intake for each vitamin (e.g., vitamin A 1^st ^month) between the control and HAD groups showed that the latter had a higher intake of the three vitamins each month during the study (p < 0.001, Mann-Whitney U-Rank Sum test).

### Peripheral blood and leukocyte concentration of vitamins

The concentration in plasma showed deficiency in three of the four vitamins analyzed. The basal peripheral blood concentration of serum α-tocopherol showed a deficiency (< 600 μg/dl) in fifteen (34.5%) and seventeen (36.1%) women in the control and HAD groups, respectively. In addition, five (11.6%) and six (12.8%) women showed a deficiency of vitamin C in plasma (< 4.0 μg/ml); meanwhile, eleven (25.6%) and ten (21.3%) women had a deficient leukocyte ascorbate concentration (20 μg/10 cells), respectively. We found neither any decrease nor any deficiency for serum vitamin A in any patient in any of the groups.

Comparisons showed that, in the control group, there were no statistical differences in any of the vitamins concentrations. On the other hand, the HAD group showed an increase in all the vitamin concentrations after the first (serum α-tocopherol; p < 0.001), second (plasma ascorbate; p < 0.001) and third months (serum retinol and leukocyte ascorbate; p < 0.001, Friedman Analysis of Variance on Ranks, Dunn's post hoc test in all cases) of intervention (Table 4).

**Table 4:** Vitamin concentrations in the control and HAD groups.

	**Serum Retinol (μM/L)**		**Serum α-tocopherol (μM/L)**		**Plasma ascorbate (μM/L)**		**Leukocyte ascorbate (μg/10^8 cells)**	
	Control	HAD	Control	HAD	Control	HAD	Control	HAD
Basal	2.0 ± 0.4	2.0 ± 0.3	14.2 ± 2.4	14.0 ± 1.8	62.7 ± 11.5	62.6 ± 10.3	27.8 ± 4.7	27.9 ± 4.8
1^st ^month	2.0 ± 0.3	2.1 ± 0.3	15.3 ± 2.3	15.8 ± 1.6*	62.8 ± 13.5	67.1 ± 9.9	28.0 ± 4.0	29.5 ± 4.1
2^nd ^month	2.1 ± 0.3	2.2 ± 0.2	14.4 ± 2.0	16.6 ± 1.8*^#^	65.6 ± 11.7	79.8 ± 10.6*^#^	26.1 ± 6.1	31.0 ± 3.9^#^
3^rd ^month	1.9 ± 0.4	2.3 ± 0.3*^#^	14.2 ± 2.2	18.1 ± 1.9*^#^	67.1 ± 11.4	96.9 ± 9.6*^#^	25.2 ± 9.9	35.8 ± 3.7*^#^
4^th ^month	2.1 ± 0.5	2.4 ± 0.4*^#^	14.3 ± 2.1	18.7 ± 1.7*^#^	67.5 ± 12.0	120.6 ± 9.8*^#^	28.7 ± 7.3	37.6 ± 3.9*^#^

Data are means ± SD. * Friedman Repeated Measures Analysis of Variance on Ranks (Dunn's post hoc test) for comparisons in the control and HAD groups (basal vs. all months). ^# ^Mann-Whitney U-Rank Sum test for comparisons in the basal and every month concentrations between the control and HAD groups. p < 0.05 in all cases.

All vitamin concentrations were statistically different between the control and HAD groups from the second (serum α-tocopherol, plasma and leukocyte ascorbate; p < 0.001, Mann-Whitney U-Rank Sum test) and third months (serum retinol; p < 0.05, Mann-Whitney U-Rank test) (Table 4).

### Antioxidant enzymes activity and oxidative stress markers

SOD and GPx enzymatic activities increased from the second and third months of intervention in the HAD group, respectively (p < 0.001, Friedman Analysis of Variance on Ranks, Dunn's post hoc test) (Table 5). This phenomenon was not observed in the control group. Comparisons of the enzymatic activities between the control and HAD groups showed that the latter had a higher SOD activity after the first month (p < 0.001, Mann-Whitney U-Rank Sum test) and a higher GPx activity in the third month (p < 0.001, Mann-Whitney U-Rank Sum test).

**Table 5:** Antioxidant enzyme activity and oxidative stress markers in the control and HAD groups.

	**Superoxide Dismutase (U/mL)**		**Glutathione Peroxidase (nmol/min/mL)**		**Malondialdehyde (μM/L)**		**Lipid Hydroperoxides (μM/L)**	
	Control	HAD	Control	HAD	Control	HAD	Control	HAD
Basal	3.9 ± 1.6	4.0 ± 1.1	1031.1 ± 114.2	1033.6 ± 108.6	31.6 ± 5.4	31.1 ± 4.7	12.3 ± 2.2	12.1 ± 3.3
1^st ^month	3.8 ± 2.0	4.8 ± 1.3^#^	1078.7 ± 119.9	1078.3 ± 105.2	30.6 ± 5.2	30.9 ± 4.2	11.9 ± 2.3	11.4 ± 3.2
2^nd ^month	4.1 ± 1.2	5.6 ± 1.8*^#^	1066.8 ± 112.2	1106.6 ± 121.7	30.4 ± 4.9	28.7 ± 4.0	11.7 ± 2.8	11.0 ± 2.9
3^rd ^month	3.9 ± 1.5	6.1 ± 2.0*^#^	1081.9 ± 108.0	1300.5 ± 111.7*^#^	31.3 ± 4.7	25.2 ± 2.3*^#^	11.5 ± 2.4	9.6 ± 2.2*^#^
4^th ^month	3.8 ± 1.4	9.2 ± 3.2*^#^	1076.6 ± 118.8	1564.3 ± 137.3*^#^	30.9 ± 3.2	23.0 ± 2.3*^&^	11.8 ± 2.1	8.2 ± 1.9*^#^

Data are means ± SD. * Friedman Repeated Measures Analysis of Variance on Ranks (Dunn's post hoc test) for comparisons in the control and HAD groups (basal vs. all months). ^# ^Mann-Whitney U-Rank Sum test for comparisons in basal and every month concentrations between the control and HAD groups. *P *< 0.05 in all cases.

The oxidative damage to lipids showed a decrease in the MDA and LPH concentrations in the third month (p < 0.001, Friedman Analysis of Variance on Ranks, Dunn's post hoc test). No decrease in any of the oxidative stress markers was found in the control group. The intergroup comparison demonstrated that the HAD group had a lower concentration of LPH and MDA in the third month of intervention (p < 0.001; Mann-Whitney U-Rank Sum test (Table 5).

## Discussion

### Women without vs. with endometriosis: Antioxidant intake

In this work, we observed and confirmed previous data regarding a lower antioxidant intake in WEN when compared with women without the disease [28]. Vitamin C, copper and zinc intakes were above the RDI in both groups. Nevertheless, WEN showed a 30% lower intake of these antioxidants in comparison to WWE. WEN did not even fulfill their vitamin E minimum intake, which was 40% less than that of WWE. A possible explanation of vitamin E deficient intake observed in WEN could be an association with nutritional customs and behavioral habits, such as the low dietary consumption of nuts, wheat germ, sunflower seeds, and extra virgin olive oil [23].

Previous studies done in the U.S. population have shown that only 8–11% of men and 2–8% of women meet the new estimated average requirement (EAR) for vitamin E, according to the Continuing Survey of Food Intakes by Individuals (CSFII,1994–1996) and the National Health and Nutrition Examination Survey (NHANES, 2001–2002) [29]. However, there is little information about α-tocopherol intake in the Mexican population, which may have different dietary patterns from the U.S. population. In those studies done in Mexico, only the plasma concentrations of α-tocopherol have been evaluated in children, 2-year-old children, men and women; the evaluations found a high prevalence of serum α-tocopherol concentration deficiency [30]. The Mexican National Nutrition Survey in 1999 showed a serum α-tocopherol deficiency of nearly 30% in non-pregnant and pregnant women. This deficiency is similar to the one that we found in women with endometriosis in the control (34.5%) and HAD (36.1%) groups. Although vitamin E deficiency is high in the Mexican population, studies of its frequency and consequences for health are very limited or nonexistent. Beside Parazzini's observations, our group [28] has reported on the antioxidant vitamins intake in women with and without endometriosis, finding that vitamin C, vitamin E, selenium and zinc intake in the women with endometriosis showed a statistical difference when compared with the control group. Selenium intake was around 60% of the RDI in both cases. This observation is in line with data reporting that selenium intake depends on the selenium content of the soil where plants are grown or animals are raised [31].

The body's complex antioxidant system is influenced by dietary intake of non-enzymatic antioxidants such as manganese, copper, selenium and zinc, beta-carotenes, vitamin C, vitamin E, taurine, hypotaurine, and B vitamins [12]. On the other hand, the body produces several antioxidant enzymes such as catalase, superoxide dismutase, glutathione reductase, glutathione peroxidase and molecules like glutathione and NADH. Glutathione, a tripeptide, is produced by the cell and plays a crucial role in maintaining the normal balance between oxidation and antioxidation. NADH is considered an antioxidant in biological systems due its high reactivity with some free radicals, its high intracellular concentrations and the fact that it has the highest reduction power of all biologically active compounds [32]. Both enzymatic and non-enzymatic antioxidant systems scavenge and deactivate excessive free radicals, helping to prevent cell damage.

### Control diet vs. HAD

After the FFQ analysis, we observed that in 5 of the 6 antioxidants analyzed, the antioxidant intake in WEN was 33% less than in WWE. Also, two nutrients (vitamin E and selenium) did not even reach their RDI. Thus, from these data, we decided to design a high antioxidant diet and evaluate its effect in WEN to improve peripheral antioxidant markers. With the development of the HAD, we made a simple and feasible diet that could guarantee the supply of the proposed values. Although they were not all reached (vitamin A: 95%; vitamin E: 66%; vitamin C: 79%), the evidence showed that this diet had a positive effect by diminishing the oxidative stress markers and improving the antioxidant enzyme activity and the vitamin concentrations in peripheral blood.

The 24-hour recalls done in every follow-up visit showed that patients duplicated their vitamin C intake (203% to 523%) through adherence to the HAD. The increment in vitamin C intake had a direct effect on its plasma and leukocyte concentration from the second and third months of study. On the other hand, although vitamin E and A intake did not duplicate these results (61% to 88%; 112% to 143%, respectively), it was possible to observe a statistically increment of serum concentration in both vitamins from the second and third months of study, respectively.

The abovementioned intakes were enough to observe a decrease in the oxidative stress markers from the third month of intervention. Studies using fruit and vegetables (as food or concentrates) in different periods of time have observed a decrease of 4% to 40% in oxidative stress markers (8-isoprostanes, LHP, MDA) depending on the amount of vitamin A, C and E supplements [33-35]. In our case, we found a decrease of 20% of MDA and LPH after three months of following the HAD.

Vitamin E is the most effective lipid-soluble, chain-breaking antioxidant, singlet oxygen quenchers against lipid peroxidation. In our study, we achieved a change of 29% and 34% in the third and fourth months with 20 mg of α-tocopherol from seeds; this is half of the dose used by Uptrichard [36] and 2.5 and 5 times less than the doses used by Pallast [37]. Uptrichard used a spread supplemented with 43 mg of α-tocopherol equivalents for almost three months (11 weeks) in healthy, non-smoking adults and observed a change of 31% in their plasmatic concentration at the end of the study. Pallast supplemented elderly people for 6 months (24 weeks) with 50 and 100 mg of DL α-tocopherol acetate and observed a 35% and 50% increase, respectively, in the plasma concentration of α-tocopherol. This effect can be attributed to the bioavailability of α-tocopherol present in the sunflower seeds and peanuts in comparison to α-tocopherol used in the spread and pills. It is well documented that natural vitamin E is twice as effective as synthetics, since the former stays for longer in tissues [38]. In addition, intake of vitamin E with vitamin C could be another reason for improvements, due to the fact that the latter can effectively turn the α-tocopheroxyl radical to α-tocopherol [39]. In 2006, Bruno et al. found that supplementation with vitamin C decreased plasma α-tocopherol disappearance rates in smokers [40].

For vitamin A, it was possible to observe an increase of 12% in its plasmatic concentration at the end of study. Several studies have found different increases (from 3% to 500%) in vitamin A concentrations (α-carotenes, β-carotenes, lycopenes) with mixed fruit and vegetable concentrate capsules, soups and beverages in healthy men and women [41,42]. We believe that this small increase in serum retinol was because women did not have serum vitamin A deficiency and their intake of this vitamin was always just above its SDI, a phenomenon not observed with vitamin E.

Plasmatic vitamin C reflects the vitamin C intake of a previous day. On the other hand, leukocyte ascorbate concentration gives us information about the vitamin C pool, which is a consequence of the vitamin C intake of 20 days before. We observed a change of 40% in plasma ascorbate from the second month and a leukocyte ascorbate change of 28% from the third month to the end of the study, respectively; confirming the positive effect of the HAD on such vitamins in the body.

On the other hand, it is known that inorganic nutrients are essential for the optimal functioning of antioxidant enzymes. In this respect, women with endometriosis showed a glutathione peroxidase activity increase of 25% from the third month of study, while superoxide dismutase activity increased by 40% after two months of study. Nevertheless, inorganic nutrients were not quantified in our study; the activity of the enzymes that we evaluated is associated with the increase of inorganic nutrients, such as selenium, zinc, magnesium, and iron.

Based on the results shown in the present manuscript, we can suggest that the follow-up of a HAD with pertinent adaptations could be used as a therapeutic aid to improve the balance of antioxidants and oxygen/nitrogen reactive species to the antioxidant side, where oxidative stress conditions persist.

## Conclusion

Women with endometriosis had lower vitamin A, C, E, zinc and copper intake in comparison to women without endometriosis.

The application of the HAD in women with endometriosis increased the peripheral concentration of vitamins A, C and E after 3 months of intervention in comparison to the control diet group.

The application of the HAD in women with endometriosis increased the peripheral enzymatic superoxide dismutase and glutathione peroxidase activity after 3 months of intervention in comparison to the control diet group.

The application of the HAD in women with endometriosis decreased the peripheral concentration of malondialdehyde and lipid hydroperoxides after 3 months of intervention in comparison to the control diet group.

## Competing interests

We declare we have no financial or other contractual agreements that might cause conflicts of interest. The corresponding author and the rest of the authors declare that have read and approved the final submitted manuscript.

## Authors' contributions

JMC participated in the coordination of the study, statistical analysis and interpretation of data, and helped to draft the study. TAS participated in the recruitment of patients, design and application of control diet and HAD, carried out the nutritional counseling and oxidative stress determinations. SBM participated in the recruitment of patients, design and application of control diet and HAD, carried out the nutritional counseling and antioxidant enzymes determinations. LJZ participated in the analysis and discussion of data. MCTL carried out the determinations of vitamins concentrations. EC carried out the interpretation and discussion of vitamins concentrations. CHG conceived the study, participated in its design, coordination, the analysis and discussion of data, and drafted the manuscript. All authors read and approved the final manuscript.
